# Supplementary material for: Sex-based differences in front crawl and butterfly sprint performance in age-group swimmers
Source: PLoS One. 2025 Dec 29;20(12):e0339480. doi: 10.1371/journal.pone.0339480 (PMC12747348; doi:10.1371/journal.pone.0339480)
Supplement: Attachment — Submitted filename: Response to Reviewers_PONE-D-25-34920docx.docx [file pone.0339480.s004.docx]

Faculty of Sport Science, Burapha University

169 Longhaad Bangsaen Road,

Chonburi, Thailand, 20131

phornpot@go.buu.ac.th

Dalton Müller Pessôa Filho, Ph.D.

Academic Editor

PLOS ONE

September 20, 2025

Subject: Revision and resubmission of manuscript PONE-D-25-34920

Dear Dr. Dalton Müller Pessôa Filho

Thank you for your letter and for providing us with the opportunity to revise our manuscript, "Determinants of Sprint in Age-Group Swimming Performance: The Role of Swimming Technique and Sex." We appreciate your and the reviewers' insightful and constructive feedback, which has been invaluable in strengthening our paper.

We have carefully considered all the points raised and have revised the manuscript accordingly. Our revisions address the concerns regarding the clarity of our methods, the statistical analysis, and the theoretical framework of our findings. We believe these changes have significantly improved the rigor and scientific contribution of our work.

We have also amended the "Role of Funder" statement to clarify the funder's role in the study. We have included the following statement in our cover letter: "The funders had no role in study design, data collection and analysis, decision to publish, or preparation of the manuscript." This statement accurately reflects that the funding body provided financial support but was not involved in the research process itself.

The following is a detailed, point-by-point response to each of the comments from the Academic Editor and reviewers. We have also included a marked-up copy of the manuscript to highlight all changes made. We hope the revised manuscript will better suit PLOS One, and we thank you for your continued interest in our research.

Best regards,

Phornpot Chainok

Faculty of Sport Science, Burapha University

169 Longhaad Bangsaen Road,

Saensuk, Mueang, Chonburi, Thailand, 20131

phornpot@go.buu.ac.th

**Response to Academic Editor Comments**

We appreciate the time and effort of the Academic Editor and the reviewers in providing constructive feedback. We have made every effort to take on board your recommendations and comments throughout the manuscript ‘Determinants of sprint in age-group swimming performance: The role of swimming technique and sex’ in response to their suggestions and hope that this improved manuscript is acceptable for publication in PLOS One. We appreciate the time and effort of the Academic Editor and the reviewers in providing constructive feedback. We have carefully considered all comments and have revised the manuscript accordingly.

**Comment 1**:

**Response 1**:

Thank you for the detailed feedback and for highlighting the specific number of self-citations in our manuscript. We acknowledge that 14 out of 40 references, while not deliberately excessive, could create the impression of a narrow focus. We would like to assure you that the number of self-citations reflects our research group's deep and specialized work in this specific area. The cited papers are not merely supplementary; they are the foundational studies that developed and validated the very methodologies and conceptual frameworks used in the present manuscript. For instance, our previous work established the specific kinematic analysis protocols that are central to this study's findings on swimming technique. Therefore, referencing them was essential for methodological transparency and to provide a complete picture of our research trajectory.

However, we agree that a broader context is vital for the manuscript's scholarly contribution. We have carefully reviewed and revised the manuscript, replaced some self-citations where suitable alternatives from other authors were available, ensuring that our work remains in conversation with the wider scientific community. We are confident that these revisions maintain the necessary link to our foundational work while also addressing your concern by integrating a more diverse body of literature. We are grateful for your guidance in helping us improve the manuscript.

**Response to Journal Requirements**

PONE-D-25-34920R1
Determinants of Sprint in Age-Group Swimming Performance:  The Role of Swimming Technique and Sex
Dr. Phornpot Chainok

Dear Dr. Chainok,

We've checked your submission and before we can proceed, we need you to address the following issues:

1.  We note that your Data Availability Statement is currently as follows: [All relevant data are within the manuscript and its Supporting Information files.]

Please confirm at this time whether or not your submission contains all raw data required to replicate the results of your study. Authors must share the “minimal data set” for their submission. PLOS defines the minimal data set to consist of the data required to replicate all study findings reported in the article, as well as related metadata and methods ([https://journals.plos.org/plosone/s/data-availability#loc-minimal-data-set-definition](https://journals.plos.org/plosone/s/data-availability#loc-minimal-data-set-definition)).

For example, authors should submit the following data:

- The values behind the means, standard deviations and other measures reported;

- The values used to build graphs;

- The points extracted from images for analysis.

Authors do not need to submit their entire data set if only a portion of the data was used in the reported study.

If your submission does not contain these data, please either upload them as Supporting Information files or deposit them to a stable, public repository and provide us with the relevant URLs, DOIs, or accession numbers. For a list of recommended repositories, please see [https://journals.plos.org/plosone/s/recommended-repositories](https://journals.plos.org/plosone/s/recommended-repositories).

If there are ethical or legal restrictions on sharing a de-identified data set, please explain them in detail (e.g., data contain potentially sensitive information, data are owned by a third-party organization, etc.) and who has imposed them (e.g., an ethics committee). Please also provide contact information for a data access committee, ethics committee, or other institutional body to which data requests may be sent. If data are owned by a third party, please indicate how others may request data access.

We've returned your manuscript to your account. Please resolve these issues and resubmit your manuscript within 21 days. If you need more time, please email the journal office at [plosone@plos.org](mailto:plosone@plos.org). We are happy to grant extensions of up to one month past this due date. If we do not hear from you within 21 days, we will withdraw your manuscript.

Please log on to PLOS Editorial Manager at [https://www.editorialmanager.com/pone/](https://www.editorialmanager.com/pone/) to access your manuscript. You will find your manuscript in the 'Submissions Sent Back to Author' link under the New Submissions menu. Be sure to remove your previous manuscript file if you are uploading a new file in response to these requests. After you've made the changes requested above, please be sure to view and approve the revised PDF after rebuilding the PDF to complete the resubmission process.

We are requesting these changes to comply with the PLOS ONE submission guidelines ([https://journals.plos.org/plosone/s/submission-guidelines](https://journals.plos.org/plosone/s/submission-guidelines)). Please note that we won't send your manuscript for review until you have resolved the above requests.

Thank you for submitting your work to PLOS ONE and supporting our mission of Open Science.


Kind regards,

Ronalyn M. Ramos
PLOS ONE

**Comment 1:** Please ensure that your manuscript meets PLOS ONE's style requirements, including those for file naming. The PLOS ONE style templates can be found at

https://journals.plos.org/plosone/s/file?id=wjVg/PLOSOne_formatting_sample_main_body.pdf and https://journals.plos.org/plosone/s/file?id=ba62/PLOSOne_formatting_sample_title_authors_affiliations.pdf

**Response 1:** Thank you for the reminder. We have carefully reviewed our manuscript against the provided PLOS ONE style templates for both the main body and the title/authors/affiliations section. We can confirm that all formatting, including headings, citations, and figure/table legends, now meets the journal's requirements. We have also ensured our files are named correctly according to the guidelines. We believe the manuscript is now ready for your review.

**Comment 2:** You indicated that you had ethical approval for your study. In your Methods section, please ensure you have also stated whether you obtained consent from parents or guardians of the minors included in the study or whether the research ethics committee or IRB specifically waived the need for their consent.

**Response 2:** Thank you for your comment. We have reviewed the Methods section and can confirm that we have included a statement regarding parental consent. As stated in the manuscript, "The participants and their guardians were provided with a comprehensive explanation of the testing protocols before obtaining signed consent for participation," confirming that we obtained written informed consent from the parents or legal guardians of all minor participants.

**Comment 3:** Thank you for stating the following financial disclosure:

“Faculty of Sport Science, Burapha University THAILAND”

Please state what role the funders took in the study. If the funders had no role, please state: "The funders had no role in study design, data collection and analysis, decision to publish, or preparation of the manuscript." If this statement is not correct you must amend it as needed.

Please include this amended Role of Funder statement in your cover letter; we will change the online submission form on your behalf.

**Response 3:** Thank you for your comment. We have amended the "Role of Funder" statement to clarify the funder's role in the study. We have included the following statement in our cover letter: "The funders had no role in study design, data collection and analysis, decision to publish, or preparation of the manuscript." This statement accurately reflects that the funding body provided financial support but was not involved in the research process itself.

**Comment 4:** We note that the grant information you provided in the ‘Funding Information’ and ‘Financial Disclosure’ sections do not match. When you resubmit, please ensure that you provide the correct grant numbers for the awards you received for your study in the ‘Funding Information’ section.

**Response 4:** Thank you for bringing this to our attention. We have clarified the funding information to ensure consistency. The Faculty of Sport Science, Burapha University THAILAND is the primary funding body, while the grant number [insert corrected grant number] is the specific award received. We have updated both the ‘Funding Information’ and ‘Financial Disclosure’ sections to reflect this accurately.

**Comment 5:** We note that your Data Availability Statement is currently as follows: [All relevant data are within the manuscript and its Supporting Information files.]

Please confirm at this time whether or not your submission contains all raw data required to replicate the results of your study. Authors must share the “minimal data set” for their submission. PLOS defines the minimal data set to consist of the data required to replicate all study findings reported in the article, as well as related metadata and methods (https://journals.plos.org/plosone/s/data-availability#loc-minimal-data-set-definition).

For example, authors should submit the following data:

- The values behind the means, standard deviations and other measures reported;

- The values used to build graphs;

- The points extracted from images for analysis.

Authors do not need to submit their entire data set if only a portion of the data was used in the reported study.

If your submission does not contain these data, please either upload them as Supporting Information files or deposit them to a stable, public repository and provide us with the relevant URLs, DOIs, or accession numbers. For a list of recommended repositories, please see <https://journals.plos.org/plosone/s/recommended-repositories>. If there are ethical or legal restrictions on sharing a de-identified data set, please explain them in detail (e.g., data contain potentially sensitive information, data are owned by a third-party organization, etc.) and who has imposed them (e.g., an ethics committee). Please also provide contact information for a data access committee, ethics committee, or other institutional body to which data requests may be sent. If data are owned by a third party, please indicate how others may request data access.

**Response 5:** Thank you for the detailed feedback. We have reviewed our manuscript and confirmed that all the raw data necessary to replicate the results are now included. We have updated our Data Availability Statement to reflect that all essential data, including the values behind the means, standard deviations, and any data used to create figures, are provided within the manuscript and its Supporting Information files. This ensures full transparency and reproducibility for all study findings. We believe our submission now meets PLOS ONE's minimal data set requirements.

**Comment 6:** Please include your tables as part of your main manuscript and remove the individual files. Please note that supplementary tables (should remain/ be uploaded) as separate "supporting information" files.

**Response 6:** Thank you for the clarification. We have incorporated all tables into the main manuscript and have removed the individual table files. We have also ensured that any supplementary tables are correctly uploaded as separate "supporting information" files.

**Comment 7:** If the reviewer comments include a recommendation to cite specific previously published works, please review and evaluate these publications to determine whether they are relevant and should be cited. There is no requirement to cite these works unless the editor has indicated otherwise.

Additional Editor Comments (if provided):

**Response 7:** Thanks for the reminder. We have reviewed the publications suggested by the reviewer and have determined that they are relevant to our study. We have now cited them in the appropriate sections of the manuscript to improve the context and discussion of our findings.

**Response to Reviewer 1**

**General comments**

**Comment 1:** The information is interesting. You need to make clear the presentation of results and provide more information on the development of the regression analysis.

**Response 1**: Thank you for your valuable feedback. We have revised the manuscript to provide greater clarity on the presentation of the results and to offer more detailed information on the development of the statistical analysis. We have expanded the "Statistical Analysis" and "Results" sections to more clearly present our findings and methods. A detailed explanation of our forward stepwise discriminant function analysis has been added to the "Statistical Analysis" section. This method was chosen to identify the minimum set of variables that could effectively differentiate between sexes for each swimming technique.

In the analysis, we initially included all variables that showed a statistically significant effect from preliminary univariate tests (Student's t-test). This approach ensured that all entered variables were relevant and meaningful predictors, while also creating a parsimonious model.

The variables entered into the analysis were Maturity offset, Height and Body Mass, Total CMJ Peak Force, Total IMTP Peak Force, Total Push-up Peak Force and Total Grip Strength Peak Force, AnCV, Average Mean Force (0-10s, 10-20s, and 20-30s), Fatigue Index, Average Velocity, Stroke Rate (SR), Stroke Length (SL), and Stroke Index (SI)

This analytical approach allowed us to identify the key predictors for each stroke. For example, the analysis found that total push-up peak force and body height were the most significant variables in discriminating between boys and girls in front crawl swimming. For the butterfly stroke, push-up force and stroke index were the key predictors.

**Comment 2 :** Why was peak force used that may present errors. Why not average force of 5 s?

**Response 2:** We chose to use peak force as a primary metric because it is a direct measure of maximal strength and explosive power, which are critical components of sprint swimming performance. In our study, peak force values were used to calculate the Dynamic Strength Index (DSI) for both the upper and lower limbs. This index is particularly relevant for analyzing a sport like sprint swimming, which demands explosive force for efficient propulsion. We acknowledge that a single peak force measurement could potentially be subject to minor errors or variability. However, we believe that the use of this metric (peak force) is justified for the following reasons;

(1) Relevance to performance: The goal of our study was to identify determinants of sprint performance. In this context, the ability to produce a high, instantaneous force (peak force) is more indicative of explosive capability than a sustained average force.

(2) Established Methodology: The use of peak force as a measure of explosive strength is a widely accepted and validated methodology in the field of sports science. Our approach aligns with existing literature on swimming performance and strength assessment. (DOI: <https://doi.org/10.1055/s-0035-1559694>)

(3) Comprehensive Analysis: While our analysis focused on peak force, we also collected and reported mean force at each 10-second interval to provide a more complete picture of the swimmers' force endurance throughout the 30-second swimming tethered force trial. This allows for a more nuanced understanding of the data.

Again, you are correct that with a larger sample size, incorporating average force could provide additional statistical power and a more comprehensive view of the determinants of swimming performance. We agree that exploring the role of average force with a larger cohort would be a worthwhile direction for future research, and we have added a note to this effect in the manuscript's discussion section.

**Comment 3:** You need to be more explanatory on the way you entered the variables in the forward regression analysis. How did you select the entered variables?

**Response 3:** Thank you for your valuable feedback. We have revised the manuscript to provide a more detailed and systematic explanation of our statistical methodology, specifically concerning the forward stepwise discriminant function analysis.

Our approach was designed to identify a parsimonious model that best explains the differences between boys and girls in specific swimming techniques. Here is a breakdown of our method:

**Step 1: Preliminary Analysis.** We began by conducting **univariate analyses** (Student's t-test) to examine the relationship between each potential predictor variable and sex. This preliminary step was crucial for identifying which variables had a statistically significant effect.

**Step 2: Selection of Entered Variables.** Only the variables that showed a **statistically significant difference** between boys and girls in the preliminary analysis were selected and entered into the **forward stepwise discriminant function analysis**. This process ensures that the model is built upon a foundation of meaningful and relevant predictors, avoiding the inclusion of non-significant variables that could clutter the model.

**Step 3: Forward Stepwise Analysis.** The forward stepwise method systematically added variables one by one to the model. At each step, the variable that best improved the model's ability to discriminate between the sexes was added. This process continued until no additional variable met the entry criteria to significantly improve the model. This systematic approach allowed us to identify the minimal set of variables that most effectively differentiated performance between boys and girls for each swimming stroke.

The variables initially included in the entry pool for the forward stepwise analysis, based on their significant univariate effect and controlling for biological maturity were including;

1. Anthropometry and body composition: Height, Body mass, Body mass index,

2. Upper and lower limbs neuromuscular performance: **Lower limbs neuromuscular** **performance** including Dominant CMJ peak force, Non-dominant CMJ peak force, Total CMJ peak force, CMJ asymmetry index, Dominant ITMP peak force, Non-dominant ITMP peak force, Total IMTP peak force, ITMP asymmetry index, Lower limb DSI, **Upper limbs neuromuscular performance** including Dominant push-up peak force, Non-dominant push-up peak force, Total push-up peak force, Push-up asymmetry index, Dominant grip strength peak force, Non-dominant grip strength peak force, Total grip strength peak force, Grip strength asymmetry index, Upper limb DSI

3. Anaerobic Critical velocity: AnCV

4. Swimming tethered force: average mean force (0-10s), average mean force (10-20s) (N), average mean force (20-30s) (N), fatigue index

5. Swimming performance: average velocity, stroke rate: SR, Stroke length: SL , Stroke index: SI

**Comment 4:** Where any differences between girls and boys in the regression analysis and the entered variables?

**Response 4:** Thank you for this essential question. The forward stepwise discriminant function analysis was specifically employed to determine the minimum set of variables that maximize the discrimination (i.e., the differences) between boys and girls for each swimming technique. The results confirmed that significant differences exist in the predictive variables between the sexes across the analyzed strokes. The final models for front crawl and butterfly highlighted different combinations of physiological and biomechanical factors that best characterize the differences between girls and boys:

1. Front crawl swimming: The discriminant model for front crawl swimming selected Total Push-up Peak Force and Body Height as the key predictors. This suggests that the main performance differences between boys and girls in front crawl are driven primarily by these two factors: upper-body explosive strength (as measured by the push-up force) and greater anthropometric advantage (height) typically observed in boys.

2. Butterfly swimming: The discriminant model for butterfly swimming selected Total Push-up Peak Force and Stroke Index (SI) as the key predictors. This indicates that for butterfly, while upper-body explosive strength remains a critical differentiator, the differences are also heavily influenced by swimming technique efficiency (as measured by SI), which is a key determinant of speed.

In summary, the discriminant analysis not only confirms significant differences between girls and boys but also shows that the most important factors explaining those differences vary depending on the swimming technique (particularly in front crawl and butterfly). This provides robust, multivariate evidence that the physiological and biomechanical determinants of sprint swimming are sex- and stroke-specific.

**Specific comments**

**Comment 1:** L148. Explain if all swimmers completed both strokes or they applied only one of the strokes. Showing different values in each table indicate different swimmers for each stroke.

**Response 1:** We apologize for the lack of clarity in the methodology section, which led to this confusion. We have revised the text (line 166-184), or the equivalent section in the revised manuscript) to explicitly state the following. All participating swimmers (both boys and girls) completed all elements of the testing protocol, which included the IMTP and CMJ tests, the tethered swimming assessment, and the swimming technique analysis for both the front crawl and butterfly strokes. The reason the values appear different for each stroke is not due to different groups of swimmers, but rather because of the reason including;

1.Stroke-specific demands: Front crawl and butterfly are biomechanically distinct strokes, requiring different muscle coordination and force application patterns. Therefore, a swimmer's kinetic and kinematic parameters (e.g., average force, stroke index) will naturally vary between the two strokes.

2.Focus of analysis: Our analysis was designed to show the determinants of performance for each specific stroke. Presenting separate results (and having separate discriminant function models) for the front crawl and butterfly is necessary to highlight the stroke-specific differences in performance determinants.

**Comment 2:** L169. Is this a physiological variable? Can you support this?

**Response 2:** Thank you for your insightful comment regarding our use of **Anaerobic Critical Velocity (AnCV)** and its classification as a physiological variable. We appreciate the opportunity to clarify this point. Yes, AnCV is considered a robust physiological variable in aquatic sports.

1.AnCV represents the highest swimming velocity that can be maintained without a continual net accumulation of blood lactate. It essentially marks the boundary between the heavy and severe exercise intensity domains. Therefore, it is a strong, performance-based indicator of an individual's **anerobic capacity** in the water.

2.While AnCV and the **Anaerobic Work Capacity (W′ or D′)** are both derived from the two-parameter critical power model, their physiological interpretations are distinct. W′ is the measure of the **finite anaerobic energy stores**, whereas AnCV represents the **velocity** sustained primarily by aerobic metabolism.

3.Our use of AnCV is well-aligned with the established theoretical framework for this variable in aquatic sports, as validated by foundational papers (such as those by Neiva et al., 2011, and others) that demonstrate its utility as a practical and direct measure of aerobic performance in swimming.

**Comment 3:** How 10- and 15-m time was recorded in a 25 m pool?

**Response 3:** Thank you for seeking clarification on the recording method for the 10m and 15m split times in the 25m pool. The times were accurately recorded using a standardized, high-reliability manual timing protocol to quantify the crucial acceleration phases. We used two Seiko S140 chronometers (Japan) for data acquisition. Two highly trained expert researchers were positioned precisely at the 10 m, 15 m and 25 markers along the pool deck. They simultaneously started their chronometers upon the start signal and stopped them as the swimmer's head passed their respective reference line. To ensure inter-observer reliability, the measurements from the two researchers for each split time were compared. The mean value of the two recorded times was used for the final analysis, but only if the difference between the two measurements was never greater than 0.20 seconds.

Beside, we have addressed this by providing a detailed description of the protocols, instruments, and procedures for each variable in the anaerobic critical velocity (AnCV) section, specifically within lines 249–262 of the revised manuscript.

**Comment 4:** L187. Why do you need BIA to measure body mas and body height? You probably need to express something else.

**Response 4:** Thank you for this excellent question. The observation that we used Bioelectrical Impedance Analysis (BIA) alongside an ultrasonic height meter to gather data for body mass and height is astute and requires clarification. The BIA was used to assess metrics such as Body Fat Percentage, Fat-Free Mass (FFM), and Total Body Water (TBW). These factors are highly relevant to our study, as FFM is an indicator of propulsive muscle mass, while body fat affects buoyancy and drag in the water. The standing height was measured using the integrated ultrasonic height meter component for superior accuracy and body mass was simultaneously evaluated by the integrated scale component of the BIA device. We have addressed this by providing a detailed description of the Anthropometry, body composition and biological maturation section, specifically within lines 186–193 of the revised manuscript.

**Comment 5:** L204-205. How was this adjusted to the height of each swimmer?

**Response 5:** The standing height of each swimmer was measured using an automated ultrasonic height meter (X-Contact 357S, Jawon Medical Co., Ltd., Korea) (Line 186-189).

**Comment 6:** L207. Are the apparatus used capable of separating the force for each one of the lower limbs?

**Response 6:** Yes, the apparatus used is capable of separating the force for each of the lower limbs. The data explicitly states:

The Countermovement Jump (CMJ) and Isometric Mid-Thigh Pull (IMTP) were evaluated utilizing the commercially available K-deltas dual force platforms (Kinvent Physio, Montpellier, France).The resulting peak force measurements extracted from the force-time curves included: dominant CMJ peak force (N), non-dominant CMJ peak force (N), total CMJ peak force (N)

The same separate measurements were obtained for the IMTP: dominant IMTP peak force (N), non-dominant IMTP peak force (N) total IMTP peak force (N)

The fact that the researchers were able to measure and report "dominant" and "non-dominant" peak forces for both tests, and subsequently calculate a unilateral strength asymmetry index (which compares the dominant and non-dominant limbs), confirms that the "K-deltas dual force platforms" were used to measure the force generated by each lower limb independently.

**Comment 7:** L225. What stroke was used for tethered swimming? Front crawl or butterfly or both?

**Response 7:** We confirm that the tethered swimming force tests were conducted for both front crawl and butterfly techniques in separate sessions, as described in the Materials and Methods section (Line 238-248).

**Comment 7:** L265. What do you mean significant effect? On what was this based? You need to name these variables in the results section

**Response 7:** By “significant effect,” we referred to variables that showed statistically significant sex differences (p < 0.05) in the MANCOVA analyses after controlling for maturity offset. Specifically, in the results section, the discriminant function analysis included variables such as total push-up peak force and body height in front crawl, and total push-up peak force and stroke index in butterfly, as these were identified as having significant discriminatory power between sexes.

**Comment 7:** L291-295. Some of these variables are interrelated and should not be used in the regression analysis. You should consider this. What was the dependent variable?

**Response 7:** We thank the reviewer for this important observation. In our analysis, only variables that showed statistically significant differences in the MANCOVA (p < 0.05) were retained for entry into the forward stepwise discriminant function analysis, thereby reducing redundancy from interrelated predictors. The dependent variable in this model was sex (boys vs. girls), while the independent variables comprised anthropometric, neuromuscular, tethered force, AnCV, and swimming performance measures. The final discriminant models identified total push-up peak force and body height in front crawl, and total push-up peak force and stroke index in butterfly, as the strongest predictors differentiating sexes. Importantly, the stepwise procedure incorporated collinearity diagnostics, ensuring that interrelated variables did not compromise the robustness of the regression results.

**Comment 8:** Table 1. Use “Body mass” instead of “weight”

**Response 8:** We appreciate the reviewer’s suggestion and have revised Table 1 accordingly, replacing the term “weight” with “body mass” to ensure consistency with scientific terminology.

**Comment 9:** Table 2. Non-dominant grip strength peak force (N) 13.27 ± 0.33 and 13.73 ± 0.96. These values are not correct. Also, the grip strength asymmetry values are missing.

**Response 9:** In Table 2, the non-dominant grip strength peak force values were inadvertently overwritten by the age values. We have corrected them to boys 113.27 ± 33.33 N ; girls: 201.62 ± 46.40 N, and added the missing Grip strength asymmetry index (%) (boys: 7.08 ± 1.27; girls: 9.02 + 1.21). The table has been updated accordingly.

**Comment 9:** Table 3. The height should be corrected.

**Response 9:** It was corrected accordingly.

**Comment 10:** Table 3 and Table 4. Similar to table 1 and 2, you should reconstruct the tables avoiding repetitions of the same values.

**Response 10:** We appreciate the reviewer’s constructive feedback. Following the same approach as with Tables 1 and 2, we have reconstructed Tables 3 and 4 to avoid unnecessary repetition of values and to improve clarity and readability. The revised tables now present the data in a more concise and non-redundant format.

**Comment 10:** Table 5. You need to provide the r of the regression

**Response 10:** We thank the reviewer for the comment. Since Table 5 presents results from a discriminant function analysis rather than a regression model, correlation coefficients (r) are not applicable to this type of analysis.

**Response to Reviewer 2**

**Comment 1:** This study investigates the determinants of sprint performance in age-group swimmers, with a particular focus on sex-based comparisons. Overall, the manuscript is well written, methodologically sound, and provides interesting insights into the role of technique and other relevant variables in sprint swimming. The scientific rigor is evident, and the paper is clearly structured. That said, I must admit I was somewhat disappointed after reading the paper in light of its title.

While the study compares boys and girls across several performance-related variables, it does not go far enough in identifying the actual determinants of sprint performance within each sex. The analysis mainly highlights in which variables boys outperform girls, but it remains unclear which specific factors are most strongly associated with performance outcomes in each group. Simply reporting that boys are generally taller, stronger, and faster than girls does not add much novelty to the literature, as these differences are relatively well established.

Maybe an intra-sex analysis to determine which variables best predict performance for boys and for girls separately could enrich the study? For example, which technical or physical measures are more decisive in explaining performance within each sex? Since boys and girls do not compete directly against each other in real settings, the practical value of focusing solely on inter-sex differences is limited. For this reason, I strongly encourage the authors to emphasize intra-sex determinants, as this would make the study more impactful and novel. Such an approach would provide more meaningful insights for both researchers and coaches, and it seems that the data needed to perform this analysis are already available.

**Response 1:** We sincerely appreciate the reviewer’s thoughtful suggestion to conduct intra-sex analyses to identify the strongest predictors of sprint performance for boys and girls separately. We agree that such an approach could indeed enrich the study and provide practical insights for coaches and practitioners. However, our sample size (n = 30; 15 boys and 15 girls) was limited to national-level age-group swimmers specializing in short-distance events. Conducting separate multivariate models within each sex would risk statistical underpowering, overfitting, and unstable estimates, which might compromise the validity of the findings. From a statistical perspective, multivariate analyses such as MANCOVA and stepwise discriminant function analysis require adequate case-to-variable ratios (commonly recommended at least 10–15 cases per predictor), which our subsamples could not satisfy. Running intra-sex models with only 15 observations per group would therefore inflate Type I error, reduce generalizability, and undermine the robustness of the explained variance estimates. For this reason, we focused our analysis on sex-based comparisons, applying univariate tests, MANCOVA, and discriminant analysis to identify the smallest set of variables that maximized inter-sex differences. At the same time, we highlighted the value of intra-sex predictors in the limitations and future directions sections, where we recommend further studies with larger samples that can properly power intra-sex models. We believe this approach ensures methodological rigor while recognizing the reviewer’s valuable suggestion for advancing this research line in the future.

**Comment 2:** The reported multivariate analyses are indeed interesting: sex differences were observed particularly in upper-body force production and stroke efficiency, and discriminant analysis identified total push-up peak force and body height as key discriminators in front crawl, while push-up force and stroke index were the main predictors in butterfly. These results are valuable, but the study could be substantially strengthened by extending this approach to identify intra-sex predictors of sprint success.

**Response 2:** We thank the reviewer for the constructive comment and for recognizing the value of our multivariate analyses in identifying sex-based differences in force production and stroke efficiency. We agree that extending the analysis to intra-sex predictors of sprint performance could provide additional insights of practical relevance. However, our sample comprised only 30 national-level age-group swimmers (15 boys and 15 girls), which limited the statistical power required for stable multivariate modeling within each sex. In particular, discriminant and stepwise regression techniques rely on adequate case-to-variable ratios (commonly ≥10–15 cases per predictor) to ensure reliable estimates and avoid model overfitting. With only 15 participants per subgroup, intra-sex analyses would likely yield unstable coefficients, inflated Type I error rates, and reduced generalizability of the findings. For this reason, we focused on inter-sex comparisons, which allowed us to conduct robust MANCOVA and discriminant analyses and report interpretable differences in technical and neuromuscular predictors across sexes. Nevertheless, we fully acknowledge the importance of intra-sex determinants and have highlighted this as a key direction for future studies with larger samples that can more appropriately address predictors of sprint success within each sex.

**Comment 3:** I would also suggest considering the inclusion of actual performance times (e.g., 50 m sprint results), as these are highly relevant for coaches and practitioners who will read the paper. Beyond statistical findings, it would be very useful to add one or two paragraphs with concrete practical applications—what should coaches take from these results in terms of training focus, technical development, or talent identification?

**Response 3:** We sincerely thank the reviewer for this valuable suggestion. In the present study, all participants were national-level age-group swimmers specializing in sprint events, and 50 m performance times were indeed collected as part of the testing protocol. However, given the small and homogeneous nature of the sample, we considered sprint time mainly as a background performance descriptor rather than as a dependent variable in the multivariate models. Moreover, from a statistical perspective, the inclusion of performance times as dependent variables in forward stepwise discriminant function analysis may introduce severe multicollinearity, as these outcomes are inherently composed of and strongly overlap with the predictive variables. This could artificially inflate the model’s explanatory power, mask the relative contribution of individual predictors, and ultimately reduce the interpretability of which technical or physical factors truly drive sprint performance.

**Comment 5:** Lines 150–154: the data mentioned here already appear in the tables and don't need to be repeated.

**Response 5:** We thank the reviewer for this observation. We agree that the data mentioned in lines 150–154 already appear in the corresponding tables. To avoid redundancy and improve clarity, we have removed the repeated values from the text and retained only the key points for interpretation.

**Comment 6:** Please clarify how the AnCV was performed. Were starts conducted in-water? Were freestyle and butterfly trials randomized?

**Response 6:** We thank the reviewer for this important comment and the opportunity to clarify the procedures related to the anaerobic critical velocity (AnCV) assessments. All trials (10 m, 15 m, and 25 m) were initiated from an in-water push start to minimize the influence of start performance on velocity measurements. The order of the freestyle and butterfly trials was randomized across participants to reduce potential sequencing or fatigue effects, with a standardized 30-minute passive recovery between techniques to ensure adequate rest. These procedures were consistent with the methodological recommendations of Neiva et al. (2011) and other studies applying the critical velocity model in swimming. We have revised the methods section to explicitly state these details for improved transparency.

**Comment 7:** Line 311: correct “From”.

**Response 7:** We thank the reviewer for pointing out this typographical error. The word “From” has been corrected accordingly.

**Comment 7:** In summary, this is an interesting and well-executed study, but in its current form it mainly confirms expected differences between sexes. A deeper analysis into intra-sex determinants of sprint swimming performance, alongside clearer practical implications for coaches, would substantially increase the value and contribution of the manuscript to the field.

**Response 7:** We sincerely thank the reviewer for the encouraging summary and for recognizing the value of our study. We fully agree that examining intra-sex determinants would provide additional insights; however, as noted previously, our sample size (15 boys and 15 girls) limited the statistical power required for stable multivariate analyses within each sex, and attempting such models would risk overfitting and unstable estimates. To address this valuable suggestion, we have emphasized intra-sex determinants as a key direction for future research with larger cohorts. In addition, we have expanded the discussion to include clearer practical applications, highlighting how the discriminative role of upper-body force production, body height, and stroke index can inform training priorities, technical development, and talent identification. These revisions aim to enhance the practical relevance of our findings while maintaining methodological rigor.
