# Supplementary material for: Sex-based differences in front crawl and butterfly sprint performance in age-group swimmers
Source: PLoS One. 2025 Dec 29;20(12):e0339480. doi: 10.1371/journal.pone.0339480 (PMC12747348; doi:10.1371/journal.pone.0339480)
Supplement: Attachment — Submitted filename: Response to Reviewers.docx [file pone.0339480.s005.docx]

**Response to Academic Editor Comments**

We sincerely appreciate the time and effort that the Academic Editor and reviewers have dedicated to providing thoughtful and constructive feedback. We have carefully considered all comments and incorporated the recommended revisions throughout the manuscript entitled “Determinants of sprint in age-group swimming performance: The role of swimming technique and sex.” We hope that the improvements made in response to these suggestions meet the expectations of the Academic Editor and reviewers and that the revised manuscript will be deemed suitable for publication in PLOS ONE.

**Comment 1**: I agree with Reviewer #1 that the AnCV term is not appropriate for the slope variable of the distance trial-time modelling in swimming. Thank you for addressing all of my other comments.

**Response 1**:

We sincerely thank the Academic Editor for the helpful clarification regarding the use of the term anaerobic critical velocity (AnCV) in the manuscript. We fully agree that the term should be used with caution when referring to the slope of the distance–time (D–t) modelling, as it may imply a direct and exclusive measurement of anaerobic metabolism. In response, we have revised the manuscript to ensure that the slope variable is described in a more physiologically neutral manner and does not unintentionally overstate its metabolic implications.

“The regression model is expressed as y = ax + b, where y denotes the distance covered and x represents time. In this context, the coefficient an is primarily recognized as the short-distance velocity coefficient (slope), while also being recognized for its conventional meaning in the literature as part of the anaerobic critical velocity (AnCV). The constant b corresponds to the y-intercept of the regression line”

Our methodological approach was originally based on the procedure proposed by Neiva et al. (2011), who introduced the AnCV framework as a performance-oriented derivative of the critical power concept applied to short-distance swimming. This framework has since been used as a modelling tool to characterize sprint capacity through the D–t slope rather than as a direct biochemical marker of anaerobic metabolism.

To further strengthen the conceptual justification and address the Editor’s concern, we have now incorporated additional literature demonstrating the scientific acceptance and practical relevance of AnCV modelling in swimming research. In particular, we have added: Ruiz-Navarro et al. (2022), who reported strong associations among anaerobic critical velocity, tethered swimming force, dry-land strength measures, and sprint swimming performance. Their findings confirm that the AnCV-derived slope is a valid and meaningful indicator of short-distance performance capacity, supporting its methodological relevance within sprint profiling.

We appreciate the Editor's insightful comment, which has helped us improve both the conceptual accuracy and clarity of the manuscript.

**Response to Reviewer**

**Reviewer 1**

**Comment 1:** Your response on AnCV is not correct. This index is not connected to sustained aerobic intensity but is connected to anaerobic characteristics, however, without a physiological meaning. Fortunately you have appropriately discussed it in the manuscript.

**Response 1**: Thank you for the important clarification regarding the interpretation of AnCV. We agree with your comment that AnCV is not an indicator of sustained aerobic intensity and, although derived from the critical-velocity framework, it reflects anaerobic-related characteristics without direct physiological meaning. We have now revised our response and ensured that this distinction is clearly and accurately represented in the manuscript. In the revised text, we (i) avoided describing AnCV as an aerobic or metabolic threshold, (ii) referred to the slope parameter primarily as the short-distance velocity coefficient, and (iii) acknowledged that its use within the literature is performance-based rather than physiologically specific. We appreciate your observation that this conceptual clarification is appropriately addressed in the manuscript and have maintained this improved framing consistently across all sections.

**Comment 2:** Table 1 and Table 2 repeat the same information in most of the rows. You need to reformat

**Response 2:** Thank you for pointing out the redundancy between Table 1 and Table 2. We agree with your observation and have revised both tables accordingly. In the updated manuscript, Table 1 now includes only the anthropometric and maturational characteristics of the participants, while Table 2 presents exclusively the swimming performance, tethered force, and velocity-modelling variables. All duplicated rows have been removed, and the tables have been reorganized to ensure that each serves a distinct purpose without repetition.

**Reviewer 2**

I appreciate the authors’ efforts in addressing the first round of comments. However, after carefully reviewing both the revised manuscript and the response letter, several important issues remain insufficiently addressed. Many of my initial concerns were acknowledged in the response document but not implemented in the manuscript, or the justifications provided do not fully resolve the underlying problems raised. My detailed comments follow:

**Response:** Thank you for your careful re-evaluation of our revised manuscript and response letter. We sincerely apologize for the discrepancies between our responses and the changes implemented in the manuscript. We appreciate your patience and constructive guidance, which has significantly improved the clarity, accuracy, and rigor of our work.

**Comment 1:** The current title (“Determinants of Sprint in Age-Group Swimming Performance: The Role of Swimming Technique and Sex”) suggests an analysis of determinants of sprint performance, including factors explaining performance within each sex. However, the authors explicitly chose not to conduct intra-sex analyses and focused exclusively on inter-sex differences. If the authors intend to focus primarily on inter-sex comparisons and differences between the two swimming techniques, I recommend adjusting the title to clearly reflect this scope and avoid misleading the readers. For example, something like “Sex-Based Differences in Front Crawl and Butterfly Sprint Performance in Age-Group swimmers” would more accurately represent the actual content of the study.

**Response 1**: Thank you for this valuable comment regarding the alignment between the manuscript title and the actual scope of the study. We agree with your assessment that the original title implied an analysis of determinants of sprint performance within each sex, which could be misleading given that the primary focus of our analyses was on sex-based differences between boys and girls rather than intra-sex determinants. In response, and to ensure complete clarity for readers, we have revised the title to accurately reflect the purpose and analytical focus of the study. The new title now emphasizes the comparison of sexes and the examination of technique-specific performance characteristics. “Sex-Based Differences in Front Crawl and Butterfly Sprint Performance in Age-Group Swimmers” Thank you for helping us improve the clarity and precision of the manuscript.

**Comment 2:** The manuscript uses “sex” and “gender” interchangeably. Given that the present study examines biological and performance-related differences, I think the most appropriate term is sex. I strongly recommend standardizing terminology throughout the text.

**Response 2:** We have carefully reviewed the entire manuscript and standardized the terminology accordingly. All instances of “gender” have been replaced with “sex” to ensure consistency and conceptual accuracy throughout the text.

**Comment 3:** The order of the freestyle and butterfly tests were not randomized? Do the authors believe that the lack of randomization could have influenced the comparison between techniques?

**Response 3:** Thank you for addressing the significant concern regarding the non-randomized sequence of freestyle and butterfly sprint tests. We recognize that randomization is generally favoured for minimizing potential order or fatigue effects. The sequence (front crawl followed by butterfly) in our study was not randomized due to safety and logistical constraints. The butterfly stroke in young swimmers necessitates increased technical and physiological requirements and presents a heightened possibility of technical difficulties when performed first.

To minimize potential order effects, swimmers received complete recovery time between trials and techniques (≥10 minutes), all efforts were maximal, and coaches observed swimmers for indications of neuromuscular or physiological tiredness before advancement. Previous studies on short sprint lengths (≤25 m) demonstrate minimal residual fatigue with adequate recovery [17, 30, 39]. Consequently, we state that the testing sequence did not significantly influence the comparisons of techniques.

**Comment 4:** The authors confirm that 50 m sprint times were collected, and line 261 confirms this information exists. Yet, these data are not presented anywhere in the manuscript. Even if sprint time is not included as a dependent variable in multivariate models, it should nevertheless be reported as a descriptive performance indicator. Including these times (for each group) is essential for contextualizing the competitive level of the sample and for enhancing the manuscript’s practical value to readers.

**Response 4:** Thank you for recognizing that the 50-meter sprint data were not clearly included in the previous version of the manuscript. We agree that providing a key performance indicator is essential to contextualize the swimmers' competitive level. In the revised manuscript, we specifically highlight the 50-m swimming velocity, which we chose as it is the most prevalent and useful statistic in sprint performance evaluations. Swimming velocity serves as a direct, standardized performance metric that facilitates comparisons among groups and aligns to current recommendations in sprint-swimming research. The velocity values have been integrated into the descriptive data and included in Table 2, facilitating proper interpretation of the sample's performance level by readers. We appreciate the reviewer's feedback, and this modification has enhanced the manuscript's clarity and practical significance.

**Comment 5:** Where any differences between girls and boys in the regression analysis and the entered variables?

**Response 5:** “We have now added a new paragraph in the Discussion section (lines 343–353) that explicitly clarifies the novel contribution of the study and explains how the inter-sex comparisons advance current understanding of performance determinants in age-group sprint swimmers.

**Comment 6:** Where any differences between girls and boys in the regression analysis and the entered variables?

**Response 6:** “We thank the reviewer for this helpful suggestion. In response, we have added two well-developed paragraphs (Line 454-477) to the Discussion providing clear and actionable guidance for coaches and researchers. These paragraphs specify training considerations for boys, girls, and general recommendations applicable to both sexes.”

**Comment 7:** Another point that deserves deeper discussion: the boys and girls in the sample differ in maturity offset. Boys are farther from their PHV yet still outperform the girls across most variables. This raises several questions: How do maturity differences influence the observed results? Would the gaps be even larger if boys and girls had similar maturity status? What is the validity of directly comparing groups (already from different sexes) with different biological maturation levels? How do these findings add new insight relative to what the literature has already established? - The manuscript needs a clearer explanation of why these comparisons are meaningful and how they should be interpreted by researchers, coaches and practitioners.

**Response 7:** Thank you for raising this important point regarding differences in maturity offset between boys and girls. We agree that biological maturation is a major determinant of performance in youth sports and that maturity status must be carefully considered when interpreting sex-based comparisons. In our sample, boys were further away from their predicted PHV, however they consistently outperformed girls in most factors. This pattern corresponds with established evidence indicating that boys generally exhibit greater absolute strength, limb length, and propulsive force prior to reaching peak height velocity, attributable to earlier divergence in neuromuscular function, hormonal conditions, and anthropometric dimension.

We recognize that the disparity in maturity may somewhat affect the extent of the observed sex differences. If boys and girls were aligned in terms of maturity status, the performance disparity would likely be exacerbated, as boys undergo significant increases in muscle mass, strength, and stroke efficiency during and subsequent to peak height velocity (PHV). To address this, we incorporated maturity offset as a covariate in the multivariate modelling, ensuring that the discriminatory variables reflect true sex-related differences beyond maturational timing. Our findings therefore highlight which physical, neuromuscular, and technical factors remain most influential after accounting for maturation, offering meaningful insight into how sex-specific sprint profiles emerge during early adolescence.

This comparison is valuable for researchers and practitioners because talent identification and training prescription in youth swimming occur in mixed-maturity and mixed-sex environments. Understanding how boys and girls differ at comparable chronological ages but not necessarily identical biological ages helps coaches contextualize expected performance gaps, avoid misinterpretation of maturational advantages as “talent,” and tailor strength and technique development pathways more appropriately.
